# Singlet Oxygen Reactions with Flavonoids. A Theoretical – Experimental Study

**DOI:** 10.1371/journal.pone.0040548

**Published:** 2012-07-10

**Authors:** Javier Morales, Germán Günther, Antonio L. Zanocco, Else Lemp

**Affiliations:** 1 Universidad de Chile, Facultad de Ciencias Químicas y Farmacéuticas, Departamento de Ciencias y Tecnología Farmacéuticas, Santiago, Chile; 2 Universidad de Chile, Facultad de Ciencias Químicas y Farmacéuticas, Departamento de Química Orgánica y Fisicoquímica, Santiago, Chile; US Naval Reseach Laboratory, United States of America

## Abstract

Detection of singlet oxygen emission, λ_max_ = 1270 nm, following laser excitation and steady-state methods were employed to measure the total reaction rate constant, k_T_, and the reactive reaction rate constant, k_r_, for the reaction between singlet oxygen and several flavonoids. Values of k_T_ determined in deuterated water, ranging from 2.4×10^7^ M^−1^s^−1^ to 13.4×10^7^ M^−1^s^−1^, for rutin and morin, respectively, and the values measured for k_r_, ranging from 2.8×10^5^ M^−1^s^−1^ to 65.7×10^5^ M^−1^s^−1^ for kaempferol and morin, respectively, being epicatechin and catechin chemically unreactive. These results indicate that all the studied flavonoids are good quenchers of singlet oxygen and could be valuable antioxidants in systems under oxidative stress, in particular if a flavonoid-rich diet was previously consumed. Analysis of the dependence of rate constant values with molecular structure in terms of global descriptors and condensed Fukui functions, resulting from electronic structure calculations, supports the formation of a charge transfer exciplex in all studied reactions. The fraction of exciplex giving reaction products evolves through a hydroperoxide and/or an endoperoxide intermediate produced by singlet oxygen attack on the double bond of the ring C of the flavonoid.

## Introduction

**Figure 1:**
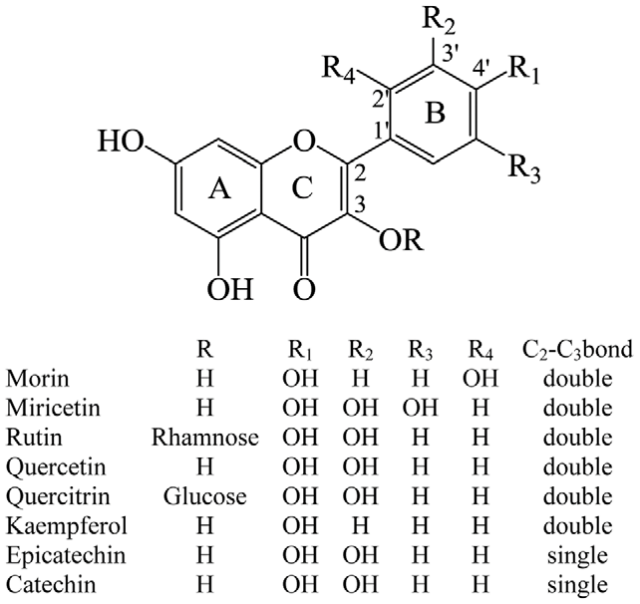
Molecular structure of studied flavonoids.

The association of Reactive Oxygen Species (ROS) to several human physiopathologies has stimulated an increasing interest in studies thereof over the last few decades. The imbalance between the production of oxidants (including ROS) and antioxidant systems (oxidative stress), is associated to several diseases among them cancer, cardiovascular diseases, and inflammatory disorders. One of the most important factors explaining the mechanism, by which these pathologies develop, involves oxidative modification of critical molecules. So, ROS can react with proteins, lipids, carbohydrates and nucleic acids, altering gene expression and promoting an inflammatory response [1]–[3]. The human organism develops protection systems against the oxidative stress, including enzymatic systems such as superoxide dismutase, glutathione peroxidases and catalases, and non-enzymatic antioxidants such as glutathione, estrogenic sex hormones and α-tocopherol. Furthermore, nowadays it is well known that exogenic antioxidants, mainly supplied by the diet, are essential to reduce the harmful effect of the oxidative stress. These antioxidants are flavonoids, phenolic acids and alcohols, stilbenes, tocopherols, tocotrienols, etc., mainly present in plants and foods. Despite major research, extensive knowledge has not yet been gained into the power of antioxidants derived from plants. Different strategies have thus been adopted to gain insight into antioxidation processes [4]–[12]. Unsaturated lipid substrates are probably prime targets of oxidation in biological environments. Lipid oxidation is a complex phenomenon induced by oxygen in the presence of initiators such as heat, free radicals, light, photosensitizers and metal ions. It occurs via three main reaction pathways: (i) nonenzymatic chain autoxidation mediated by free radicals, (ii) nonenzymatic and nonradical photooxidation, and (iii) enzymatic oxidation. The first two types of oxidation consist of a combination of reactions involving triplet oxygen, ^3^O_2_, which could be considered as a ground-state biradical OO, and singlet oxygen, O_2_(^1^Δ_g_), which corresponds to the first excited state of the molecule. There are many sources of O_2_(^1^Δ_g_) but its presence is often coupled with UV-VIS irradiation and the presence of photosensitizers. In addition, singlet oxygen in biological systems can also be formed by a chemical process which takes place in neutrophils [13]. Singlet oxygen reactions are important in biological systems, where in addition to its deleterious role (damaging valuable biomolecules) can also play beneficial roles such as in photodynamic therapy [14], [15]. One the most important family of exogenous antioxidants is the flavonoid family. The mechanism by which the flavonoids carry out their antioxidant activity has not been fully elucidated yet, in spite of the large series of studies performed. The majority of these studies have been performed empirically and only in recent times the theoretical approach has been addressed, then, many controversies remain unresolved. In particular, the specific reaction mechanism for several pairs ROS-antioxidant is not completely elucidated because a more deep knowledge on the structure–properties relationship is required. In fact, the majority of the problems appear when the antioxidant activity of flavonoids against singlet oxygen is analyzed, due to the lack of information on the intrinsic reactivity of the whole antioxidant molecule and of each ring [16]. For example, for the photosensitized oxidation of flavonoids has been suggested a reaction mechanism involving a hydroperoxide intermediate formed by singlet oxygen attack to the double bond of ring C in a like-ene reaction, or an endoperoxide intermediate produced by [2+2]-cycloaddition of the singlet oxygen to the double bond of the same ring [17], [18]. However, these intermediates have been proposed mainly considering the analysis of reaction products and not correlations between theoretical parameters that account for the molecular charge density and empirically obtained kinetic data. The methods of computational chemistry are a very helpful tool for calculate electronic properties and chemical reactivity indexes to be related with experimental measurements. Computational chemistry has the advantage of being specific, rapid and low cost, then, the aforementioned properties can be studied. In this work we report the results of an experimental and theoretical study of the reaction between singlet oxygen and a series of antioxidants of the flavonoid family, Figure 1, with the aim of generating quantitative information that allows getting a better understanding of antioxidant activity of flavonoids. We consider several structural requirements in the molecules to be studied that allow us to get a more detailed picture of the flavonoid reactivity towards singlet oxygen. These requirements are: (i) the presence of a catechol moiety in the B-ring; (ii) presence or absence of a 2,3-double bond in conjugation with a 4-oxo function in the C-ring; (iii) the substitution of the hydrogen of the hydroxyl group in position 3 of the C-ring by a bulky sugar derivative. We expect that 2,3-double bond charge density depends on the position and the number of the hydroxyl substituents on the B-ring. Also, the double bond presence is a requirement to observe the chemical reaction. In addition, we can also establish differences in the rate of physical quenching when the double bond is not present. On the other hand, voluminous substituents at position 3 of the C-ring allow us to determine the importance of steric factors in the vicinity of the reactive center. Finally, several intramolecular hydrogen bond interactions are likely. Effects on reactivity can be due to the substitution of the hydroxylic hydrogen at position 3 of C-ring, and the possible interaction between the oxygen atom at position 1 of C-ring with the hydroxyl group on position 2′ of B-ring.

## Results and Discussion

### Kinetic measurements

In homogeneous media, singlet oxygen reaction with flavonoids, F, can involve physical (deactivation) and/or chemical (reactive) processes, as is represented in the simplified mechanism (Scheme 1):

Scheme 1



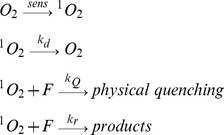
where k_d_ is the solvent dependent decay rate constant of singlet oxygen that determines its intrinsic lifetime in a given solvent (τ_o_ = 1/k_d_), k_Q_ is the second-order rate constant of the physical quenching, and k_r_ is the second-order rate constant of the reactive pathway.

Values of k_T_ ( = k_Q_ + k_r_), the total (physical and chemical) quenching rate constant and k_r_ for reaction of singlet oxygen with several flavonoids have beeen previously measured by Oliveros et al. [18] and by Mukai et al. [19], [20] (see Table 1).

**Table 1:** Values of total reaction rate constant, k_T_, and reactive reaction rate constant, k_r_, for reaction between singlet oxygen and several flavonoids.

Compound	k_r_/10^5^ M^−1^s^1^ EtOH	k_T_/10^7^ M^−1^s^1^ D_2_O, pD = 7.4	k_r_/ k_T_ (a) %	k_r_/10^5^ M^−1^ s^1^ MeOH(b)	k_T_/(M^−1^s^1^) CD_3_OD
Morin	65.7	13.4	4.9		
Miricetin	7.3		–		5.12×10^8^ (c)
Rutin	0.58	2.4	0.24	1.1	1.60×10^6(b)^ 1.21×10^8(c)^
Quercetin	5.7	5. 7	1	8.9	2.40×10^6(b)^ 4.57×10^8^ (c)
Quercitrin	0.52	2.7	0.19		
Kaempferol	2.8		–	4.8	7.10×10^5^ (b)
Epicatechin	<0.01	5.5	<0.002		1.32×10^7^ (d)
Catechin	<0.01	5.1	<0.002		5.80×10^6^ (b) 1.09×10^7^ (d)

(a) k_r_/k_T_ values calculated from data obtained in this work.

(b) Values from reference 18.

(c) Values from reference 20.

(d) Values from reference 19.

**Figure 2:**
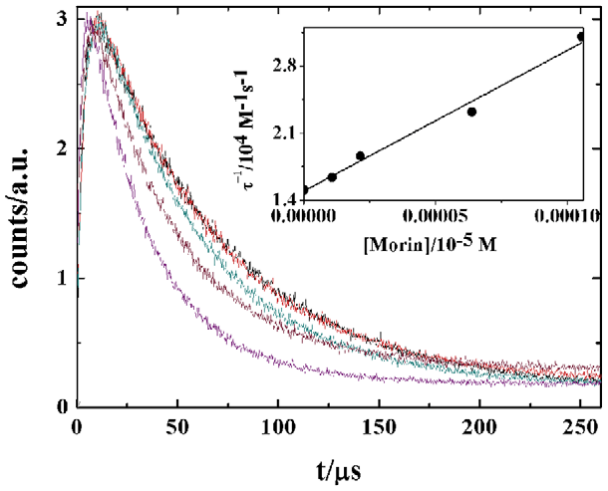
Singlet oxygen decays in the absence and the presence of morin. Rose Bengal as sensitizer, D_2_O as solvent. Inset: Stern-Volmer plot according to equation (1).

In this work, the total (physical and chemical) quenching rate constant, k_T_, for the reaction of O_2_(^1^Δ_g_) with flavonoids in D_2_O, pD = 7, as solvent were obtained from the experimentally measured first order decay of O_2_(^1^Δ_g_) luminescence at 1270 nm in the absence (τ_o_^−1^) and the presence of the flavonoid (τ^−1^). Figure 2 shows the singlet oxygen decays obtained in D_2_O with rose bengal, RB, as sensitizer, in the absence and in the presence of Morin. All decays fit a first order kinetic from which singlet oxygen lifetimes were computed. The k_T_ values were calculated from the slope of the Stern-Volmer plots according to equation (1):
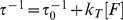
(1)where τ_0_^−1^ is the singlet oxygen lifetime in the absence of quencher and is dependent only of the solvent (τ_0_^−1^ = 1/k_d_). The observed singlet oxygen lifetime in presence of flavonoid, τ^−1^ depends on both, the solvent and flavonoid concentration. Linear plots of τ^−1^ vs. [Flavonoid] were obtained for all flavonoids in all solvents employed (Inset Figure 2). Intercept of these plots match closely with reported and measured values (in a large number of independent experiments from our laboratory) of singlet oxygen lifetime in the pure solvent, i.e. from inset of Figure 2, τ_0_ = 64 μs for D_2_O. Values of k_T_ calculated from slopes of these plots are given in Table 1. The k_T_ values were independent on the laser pulse energy (between 2 and 5 μJ) allowing us to disregard secondary processes involving O_2_(^1^Δ_g_). Traces of singlet oxygen decay after dye laser excitation at 532 nm, with Bengal Rose as sensitizer (Figure 2), in absence and presence of the flavonoid, have practically the same amplitude, indicating that excited states of the sensitizer are not deactivated by the addition of the flavonoid at the concentrations employed.

**Figure 3:**
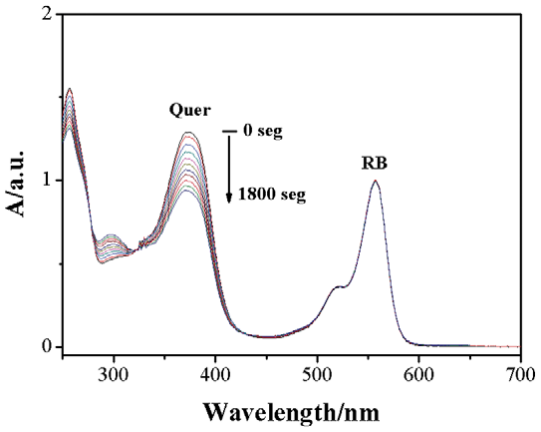
Evolution el absorption spectra of quercetin upon irradiation with visible light >500 nm in ethanol with Rose Bengal as sensitizer.

Values of k_r_ were obtained in ethanol as solvent and using Rose Bengal as sensitizer. For these experiments we select ethanol as solvent due to the low solubility of the flavonoids in water. In addition, we expect values of k_r_ around two orders of magnitude smaller than the corresponding k_T_ value, then, to get interference free UV-Vis spectra of the flavonoid and to observe a reasonable consumption of the substrate in the sensitized photoreaction we need larger concentrations of flavonoids, obtainable only in ethanol. In addition we anticipate a low solvent effect on the reaction rate if the chemical reaction involves a concerted or a partially concerted cycloaddition of the singlet oxygen on the double bond of ring C. Irradiation of aerated solutions of flavonoids in ethanol, in the presence of Rose Bengal, at the wavelength where only the sensitizer absorbs (>500 nm), decreases the concentration of the flavonoid, Figure 3.

Lineal plots of ln [flavonoid] vs. t were obtained indicating that photooxidation reaction follows a pseudo first-order kinetics, Eq. (2).
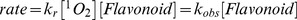
(2)


If a compound of well-known reactivity towards singlet oxygen, such as 9,10-dimethylanthracene (DMA) is employed as actinometer to evaluate the steady-state concentration of O_2_ (^1^Δ_g_) [21], the reactive rate constant for flavonoids can be easily evaluated from Eq. (3):
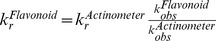
(3)


For epicatechin and catechin, we found neither substrate consumption nor product formation when monitoring the photosensitized oxidation of these compounds up to 6 h of irradiation − in ethanol as solvent − by employing spectrophotometric detection. With a careful control of experimental conditions (photon flux, temperature and cell geometry) we measured a singlet oxygen steady-state concentration equal to 1.2×10^−10^ M with DMA as actinometer. This result implies that k_r_ values for epicatechin and catechin would be ≤10^3^ M^−1^ s^−1^, then for these compounds chemical contribution to the singlet oxygen quenching is negligible.

Analysis of data in Table 1 shows that total quenching rate constants, k_T_, for reactions of all studied flavonoids with singlet oxygen are nearly independent on the flavonoid structure except for the glycoside derivatives, in which k_T_ values are a factor five smaller than the value measured for morin.

The data for k_T_ appear to be compatible with a reaction mechanism involving an exciplex formation through a charge transfer interaction between the electrophilic singlet oxygen and the π system of flavonoids, which also explains the similarity of the measured k_T_ values. Noteworthy, catechin and epicatechin quench singlet oxygen with rates comparable to the other flavonoids, in spite of catechin and epicatechin do not react chemically with singlet oxygen, giving a further support for the singlet oxygen-flavonoid-π system interaction. From the charge-transfer exciplex, the system yields products through the chemical pathway or goes through intersystem crossing to produce the parent flavonoid and ground state oxygen (Scheme 2).

Scheme 2



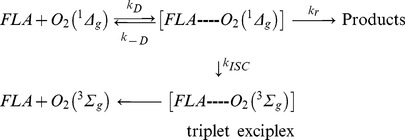



In scheme 2, k_D_ and k_-D_ are the rate constants accounting for the formation of the charge-transfer exciplex and the back reaction, respectively. A rate constant is not assigned to the dissociation of the triplet exciplex, as this is expected to be fast and the previous step is irreversible. Both, the triplet exciplex dissociation and the intersystem crossing (k_ISC_) accounts for the physical quenching channel.

The values of k_r_ obtained in ethanol as solvent, included in Table 1, are in the order of the data reported by Oliveros et al. in methanol [18]. The values of k_r_ in Table 1, indicate that chemical reactivity depends on the electronic density in the 2–3 double bond of the C-ring and the steric effects in this region of the molecule. Indeed, catechin and epicatechin as was aforementioned do not react chemically due to the lack of the double bond in the ring C. Furthermore, total rate constant values were measured for quercitrin and rutin in which the hydroxyl group in position 3 of C-ring is substituted with rhamnose and glucose, respectively. In these compounds, the presence of a bulk substituent on this position may partially block the singlet oxygen attack to the double bond diminishing reactivity of these flavonoids.

### Theoretical study

The density functional theory [22], [23] allows to employ global descriptors of electronic structure such as the electronic chemical potential and chemical hardness and softness to analyze chemical reactivity. In the framework of the Koopman theorem, chemical potential, μ (Eq. 2), and global hardness, η (Eq. 3), are easily defined in terms of the frontier molecule orbital energies (E-HOMO and E-LUMO), which represent the energy of the highest occupied molecular orbital and the energy of the lowest unoccupied molecular orbital, respectively. In addition, the global softness, S (Eq. 4), is defined as 0.5 times the inverse of global hardness and the electrophilicity index, ω (Eq. 5), a measure of the energy lowering of a ligand due to maximal electron flow between donor and acceptor, is defined as the square of its chemical potential divided by its chemical hardness. Also, the ionization potential, I, is associated with the negative of HOMO energy and the electronic affinity, A, with the negative of LUMO energy.
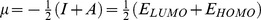
(2)

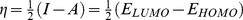
(3)

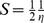
(4)

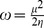
(5)


On the other hand, properties of interest for chemists, related with atoms or functional groups, rather than properties associated with points in space, are known as condensed properties [24]–[26]. The local indicator of reactivity called the condensed Fukui function, f_k_^+/−^, accounts for the nucleophilicity and electrophilicity at different sites of the molecule. The local Fukui function is a measurement of the sensitiveness of the chemical potential to changes in the external potential at a particular point k of the molecule, maintaining constant the number of electrons [27]. Another local descriptor is the local softness, s, condensed to k atom and is defined as the product of the condensed Fukui function f_k_^+/−^ times the global softness S. Thus, this descriptor includes additional information in respect of condensed Fukui function given by global molecular softness.

With the aim of clarifying the dependence of the reactivity on the electronic distribution in the flavonoid, we try to connect the values of k_T_ and k_r_ measured for their reaction with singlet oxygen, with global charge transfer properties and condensed Fukui functions. To do this, we performed electronic structure calculations using the Gaussian 09W program [28]. The geometry of all of flavonoids was optimized using the B3LYP density functional method and 6–311G+d,p basis set. A short routine reads FMO coefficients and overlap matrix to compute orbital components of Fukui function and performs the calculation of properties related with chemical potential, and condensed-to-atom Fukui functions [25]. Calculated global indexes are included in Table 2 whereas local indexes are included in Table 3. In order to avoid increase excessively the computational cost we performed the structure optimization and Fukui coefficients calculations in gas phase. However, we previously checked the effect of the media on these calculations using the simple PCM (polarizable continuums model) approach to include solvent effect. Results obtained for morin in water and ethanol are included in Table 3 and show that Fukui coefficients in these solvents do not change meaningfully and gas phase results are fully valid.

**Table 2:** Global properties related with chemical potential obtained for optimized flavonoid structure.

Compound	μ/eV	η/eV	ω/eV	S/eV^−1^	E_HOMO _/eV	E_LUMO _/eV
Morin	−3.7156	4.0417	1.70791	0.12371	−7.7573	0.3261
Miricetin	−3.8255	3.7184	1.96784	0.13447	−7.5439	−0.1071
Rutin	−3.2579	4.4907	2.36239	0.11134	−7.7486	−0.2549
Quercetin	−4.4544	4.1995	1.92959	0.11906	−8.6539	−0.017
Quercitrin	−3.7919	3.9261	1.18177	0.12735	−7.1840	1.2328
Kaempferol	−3.8421	3.8251	1.83114	0.13072	−7.6672	0.1342
Epicatechin	−4.0556	4.5225	1.81845	0.11056	−8.5781	0.4669
Catechin	−3.8975	4.3293	1.75438	0.11549	−8.2268	0.4318

**Table 3:** Fukui indexes of flavonoids molecules.

Compound	k_r_/10^5^ M^−1^s^1^ EtOH	k_T_/10^7^ M^−1^s^1^ D_2_O, pD = 7.4	f_k_^−^(C-2)	f_k_^−^(C-3)	s^−^(C-2)	s^−^(C-3)
Morin	65.7	13.4	0.1699 0.1608^a^ 0,1665^b^	0.1792 0.1626^a^ 0.1764^b^	0.02102	0.02217
Miricetin	7.3		0.0899	0.1682	0.01121	0.02226
Rutin	0.58	2.4	0.1293	0.2178	0.01464	0.02425
Quercetin	5.7	5. 7	0.0730	0.2333	0.00869	0.02778
Quercitrin	0.52	2.7	0.1003	0.1924	0.01277	0.02450
Kaempferol	2.8		0.1120	0.1937	0.01464	0.02532
Epitechin	<0.01	5.5	0.0155	0.0191	0.00171	0.00211
Catechin	<0.01	5.1	0.0241	0.0130	0.00278	0.00381

a water as solvent with PCM approach to model the media effect.

b methanol as solvent with PCM approach to model the media effect.

**Figure 4:**
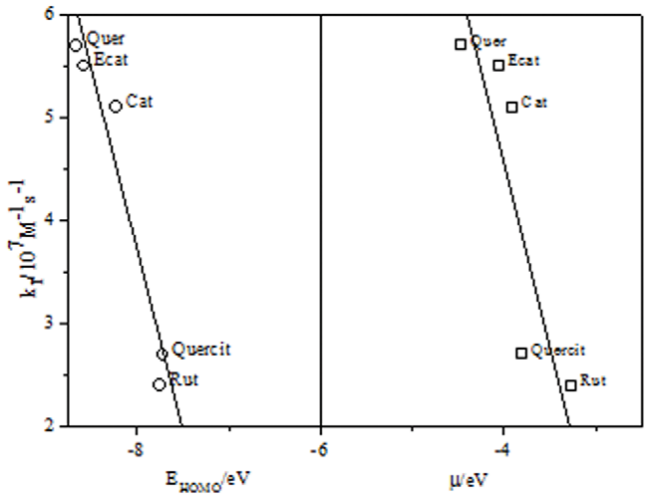
Dependence of k_T_ for reaction of singlet oxygen with flavonoids with HOMO energy and chemical potential.

Data in Table 2 are in the order of those obtained for catechin and epicatechin [29], quercetin [30] and rutin [31]. The differences of 10–15% with previously reported values may be due to the different orbital base employed in this work. Values included in Table 2, show that kinetic data obtained for reaction of flavonoids with singlet oxygen can be related to global and/or local descriptors obtained in the framework of the Koopman's approximation. Accordingly, we try to correlate the k_T_ values with global properties derived from chemical potential. As can be seen from data in Table 2, the values for all variables associated with chemical potential are low and similar. For this reason, it can be concluded that these flavonoids have the tendency to give electrons instead of capturing them. This result is an evident indication of their antioxidant ability. Also these data show that there are three groups of molecules displaying a different behavior. Morin is clearly an exception because shows an unexpected higher reactivity in spite of its variables associated to the chemical potential are close to the calculated for the other flavonoids, this particular behavior will be discussed later. Then a group with similar reactivity that includes quercetin, epicatechin and catechin, these flavonoids do not have a substituent in the hydroxyl group in position 3 of the ring C and probably represents the predictable behavior for a large family of structurally related compounds such as miricetin, kaemperol, taxifolin, apigenin, etc. Finally a third group of compounds, including rutin and quercitrin, have a bulk sugar substituent in the hydroxyl group in position 3 of the ring C, being representative of C-glycosylated flavonoids, typically present in biological systems. Figure 4 shows that values of k_T_ follow an inverse lineal dependence on both the Homo energy and the chemical potential if data for morin are omitted. Clearly this dependence indicates the presence of charge transfer interaction in which flavonoid is the electron donor, as is reflected by the increase of the k_T_ values when both Homo energy and chemical potential decrease. These dependences also explain the values of k_T_ measured for catechin and epicatechin, 5.1×10^7^ M^−1^s^−1^ and 5.7×10^7^ M^−1^s^−1^, respectively, very close to the value determined for quercetin, k_T_ = 5.7×10^7^ M^−1^s^−1^, the most recurrently studied flavonoid antioxidant. Furthermore, we consider that catechin and epicatechin have better protective effect than the other flavonoid studied here because they do not react chemically due to the absence of the double bond in the ring C. In addition to electronic effects, values of k_T_ are also dependent on the flavonoid steric hindrance when bulky substituent are present, although these effects appear to be less important than electronic ones. Accordingly, results of Table 2 and Figure 4, show that the lower reactivity was measured for rutin (k_T_ = 2.4×10^7^ M^−1^s^−1^) and quercitrin (k_T_ = 2.7×10^7^ M^−1^s^−1^). In both flavonoids hydroxyl group in position 3 of the ring C is substituted by glucose and rhamnose residues, respectively. These voluminous substituents partially block singlet oxygen–flavonoid interaction diminishing the rate constant approximately in a factor ten when compared with the non-substituted flavonoids in the same position, quercetin, epicatechin and catechin.

In order to analyze the dependence of chemical reactivity with charge density on each atom, and considering that singlet oxygen attack sites with high electron density, in Table 3 we included values of condensed Fukui function for electrophilic attack, f_k_^−^, on the C-2 and C-3 of the ring C of the flavonoid, linked by a double bond. This is the reactive site that has the largest values of f_k_^−^ of the whole molecule. In addition we included the local softness values for electrophilic attack.

**Figure 5:**
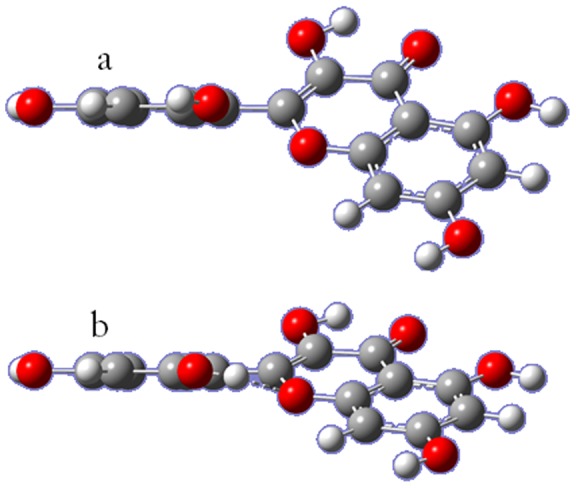
Optimized structures of morin a) obtained with DFT-6311g+d,p; b) obtained with DFT-6311g+d,p, fixing an hydrogen bond.

A complex dependence between these values and k_r_ values was observed, although the four local indexes follow the same trend as can be concluded from the analysis of Table 3. It can be observed that the lowest values of f_k_^−^ correspond to catechin and epicatechin, compounds which have reactive rate constant <1×10^3^ M^−1^s^−1^, and can be considered chemically unreactive towards singlet oxygen due to the absence of the double bond in the ring C, the reactive site. The second group of compounds have k_r_ values ranging between 2.8×10^5^ M^−1^s^−1^ and 7.3×10^5^ M^−1^s^−1^, for which around of 1–2% of the exciplex evolves through the reactive pathway to yield reaction products and morin, which deviates from any relationship. Furthermore, the largest f_k_^−^ values correspond to C-3 being probably the preferred site for singlet oxygen attack. Although our results do not constitute a definitive evidence to determine if the reaction mechanism between flavonoids and singlet oxygen corresponds to the mechanism proposed by Matsuura and co-workers [17], involving a hydroperoxide intermediate formed by singlet oxygen attack to the C-3 atom of the double bond of ring C in a like-ene reaction, or proceeds through of an endoperoxide intermediate produced by [2+2]-cycloaddition of the singlet oxygen to the double bond [18], values of f_k_^-^ for C-2 and C-3 allow us to suggest a partially concerted [2+2]-cycloaddition to form the endoperoxide. The surprisingly high reactivity of morin has no explanation in terms of global and/or local descriptors of electronic structure. Calculations of the minimized molecular structure in gas phase allow us to understand why morin shows the higher k_T_ and k_r_ values. All flavonoids have a minimum energy structure in gas phase in which the ring B plane aparts considerably from the plane of condensed rings A and C. The angle found for morin is 43.8° as can be seen in Figure 5a. It is evident that geometry prevents transmission of resonant electronic effects from ring B to the double bond. The lack of coplanarity between the π system of ring B and the double bond is common for all studied flavonoids and determinant in the reactivity.

However, morin is the only compound of the series with a hydroxyl substituent in position 2′ of ring B. This hydroxyl group could interact with oxygen in position 1 of ring C forming an intramolecular hydrogen bond through a stable six-member ring. If we fix this interaction for the minimized structure, the dihedral angle diminishes to 26.6°, Figure 5b, allowing a more effective transmission of electronic effects from ring B to the double bond. This more favorable geometry could explain the unexpected increased reactivity of morin towards singlet oxygen, however at present we have not been able to obtain the local Fukui functions of the structure with intramolecular hydrogen bond because our calculation method fails when weak interactions, such as hydrogen bonds, are included.

The large values of k_T_ observed for all the studied flavonoids indicate that they are good quenchers of singlet oxygen and could be efficient antioxidants in systems under oxidative stress. Studies in recent years [32], [33] have documented widely the higher flavonoid reactivity with radicals, antioxidant activity that can be classified as direct action. However, several considerations argue in opposition to a direct antioxidative effect in vivo: low concentration of flavonoids in the systemic circulation and in tissues, the high level of metabolism and biotransformation, flavonoid levels in the organism are transient, requiring a permanent consumption of flavonoid-rich foods to sustain a steady state concentration. These factors make the scavenging of radicals and or singlet molecular oxygen by flavonoids of very limited relevance. If we employ a roughly approximation to estimate the biological relevance of singlet oxygen quenching by flavonoids, and using a human plasma concentration for quercetin equal to 373 nM [34], the rate of disappearance of singlet oxygen, -R_SO_, would be 21.2 [O_2_(^1^Δ_g_)] s^−1^, noteworthy smaller than the predicted when reactive species are plasma protein (-R_SO_ equals to 6.6×10^5^ s^−1^[O_2_(^1^Δ_g_)]) or α-tocopherol (-R_SO_ equals to 6.2×10^3^ s^−1^ [O_2_(^1^Δ_g_)]) [35]. Anyway, actually it is unclear which endogenous antioxidants are responsible for biological system defence against singlet oxygen, because most of plasma proteins are damaged by singlet oxygen and are targets rather than antioxidants. Flavonoids such as quercetin have the advantage that only 1% of molecules react chemically with singlet oxygen, then, its steady state concentration can remain constant for a more extended time period, although lower than 7 hours [34].

### Concluding remarks

Analysis of the dependence of kinetics parameters, namely k_T_ (total rate constant) and k_r_ (chemical rate constant) obtained for reaction of flavonoids with singlet oxygen, in terms of theoretical global descriptors of electronic structure and condensed to-atom Fukui functions allows us to account for the effect of the electronic structure of flavonoids on the reaction rate and to depict the nature of the excited state encounter complex produced in the initial steps of reaction. Analysis in terms of global descriptors supports the formation of a charge transfer exciplex in all studied reactions that mainly give the parent compounds in the ground state through physical quenching. Only 1–5% of exciplex evolves to give reaction products, through an hydroperoxyde or endoperoxide intermediates produced by singlet oxygen attack on the double bond of ring C of the flavonoid, as was established from the examination of the chemical reactivity on the condensed Fukui functions. Although the relation between empirical kinetic parameters and theoretical indexes are a valuable tool to propose a reaction mechanism, a more deep understanding of the reaction can be achieved from theoretical calculations of the potential energy surface and modeling the critical complexes and intermediates that yield the final products. We look forward this open path will be closed in the near future.

## Materials and Methods

All solvents used in spectroscopic and kinetic measurements were spectroscopic, or HPLC quality. Rose Bengal (RB) (Merck), 9,10-dimethylanthracene (DMA), (Aldrich) and morin (Mor), quercetin (Quer), miricetin (Mir) quercitrin (Quercit), rutin (Rut), kaempferol (Kaem), catechin (Cat) and epicatechin (Ecat) (Sigma) were used as received.

Time-resolved luminescence measurements were carried out in 0.5 and/or 1 cm path fluorescence cells at 20°C. The sensitizer, RB, was excited with the second harmonic 6-ns light pulse of a Nd-YAG laser (532 nm, ca. 10 mJ per pulse). A liquid-nitrogen cooled North Coast model EO-817P germanium photodiode detector with a built-in preamplifier was used to detect infrared radiation emitted from the cell. The detector was at a right-angle to the cell. An interference filter (1270 nm, Spectrogon US, Inc.) and a cut-off filter (995 nm, Andover Corp.) were the only elements between the cell face and the diode cover plate. Preamplifier output was fed into the 1 MΩ input of a digitizing oscilloscope Hewlett Packard model 54540 A. Computerized experiment control, data acquisition and analysis were performed with LabView based software developed in our laboratory. Alternatively, Near-IR phosphorescence of O_2_(^1^Δ_g_) was detected by means of a customized PicoQuant Fluotime 200 system. Briefly, a diode-pumped pulsed Nd:YAG laser (FTSS355-Q3, Crystal Laser, Berlin, Germany) working at

1 kHz repetition rate at 532 nm (2 μJ per pulse) was used for excitation. The irradiated spot was aprox 7 mm^2^. The luminescence exiting from the side of the sample was filtered by an 1100nm High Performance Longpass Filter (Edmund Optics, York, U.K.) and a narrow bandpass filter at 1270 nm (NB-1270-010, Spectrogon, Sweden) to remove any scattered laser radiation. A plano-convex lens 25mm dia. ×75mm Fl, NIR II coating (Edmund Optics, York, U.K.) was used to focusing the emitted luminescence on the window of a near-IR sensitive photomultiplier tube assembly (H1033A-45, Hamamatsu Photonics Hamamatsu City, Japan) used as detector. Photon counting was achieved with a multichannel scaler (PicoQuant's Nanoharp 250). Time-resolved emission signals were analyzed using the PicoQuant FluoFit 4.0 data analysis software to extract lifetime values.

Chemical reaction rate constants were determined in ethanol using a 10 ml double wall cell, light-protected by black paint. A centered window allowed irradiation of Rose Bengal employed as sensitizer with visible light by using a 500 nm Schott cut-off filter and a visible, 50 W, Par Lamp. This setup allows to restrict light absorption only to the sensitizer. Circulating water maintained cell temperature at 20±0.5°C. A Unicam UV-4 spectrophotometer was employed to monitor the flavonoid consumption. The same experimental setup was used in parallel to measure steady state singlet oxygen concentration by employing 9,10-dimethylanthracene as actinometer.
